# Unveiling the invisible culprit: a multimodality approach for myocardial infarction with non-obstructive coronary arteries diagnosis—a case report

**DOI:** 10.1093/ehjcr/ytaf173

**Published:** 2025-04-08

**Authors:** Riccardo Terzi, Pasquale Paolisso, Francesca Di Lenarda, Edoardo Conte, Emanuele Gallinoro

**Affiliations:** Division of University Cardiology, IRCCS Ospedale Galeazzi Sant'Ambrogio, Via Cristina Belgioioso 173, 20157 Milan, Italy; Division of University Cardiology, IRCCS Ospedale Galeazzi Sant'Ambrogio, Via Cristina Belgioioso 173, 20157 Milan, Italy; Division of University Cardiology, IRCCS Ospedale Galeazzi Sant'Ambrogio, Via Cristina Belgioioso 173, 20157 Milan, Italy; Division of University Cardiology, IRCCS Ospedale Galeazzi Sant'Ambrogio, Via Cristina Belgioioso 173, 20157 Milan, Italy; Division of University Cardiology, IRCCS Ospedale Galeazzi Sant'Ambrogio, Via Cristina Belgioioso 173, 20157 Milan, Italy

**Keywords:** MINOCA, Case report, Coronary microvascular dysfunction, IMR, Vasospasm, Cardiac magnetic resonance

## Abstract

**Background:**

Myocardial infarction with non-obstructive coronary arteries (MINOCA) affects up to 10% of patients undergoing coronary angiography for acute myocardial infarction. Despite the lack of obstructive lesions, these patients face significant risks, requiring thorough diagnostic evaluations, often using both invasive and non-invasive methods. Recent guidelines emphasize the importance of performing intravascular imaging, coronary functional testing (CFT), and cardiac magnetic resonance (CMR) in the working diagnosis of MINOCA.

**Case summary:**

A 48-year-old woman presented with chest pain, elevated cardiac troponins, and signs of non-ST-elevation myocardial infarction. Echocardiography showed normal left ventricular ejection fraction with focal inferior-lateral mid-apical hypokinesia and no significant valvular heart disease. Coronary angiography revealed normal arteries with hypoplasia of the right coronary artery. Further testing, including CMR and CFT with acetylcholine provocation, confirmed severe coronary spasm, diagnosing epicardial vasospastic angina causing MINOCA.

**Discussion:**

Diagnosing MINOCA is challenging and requires a multimodal approach. Invasive testing can reveal vasospasm or microvascular dysfunction. This case emphasizes the need for repeated imaging and functional tests to reach a diagnosis. Calcium channel blockers like diltiazem are commonly used in treatment.

**Learning points**

- A multimodality diagnostic approach combining coronary angiography, cardiac MRI, and coronary functional testing is pivotal in diagnosing MINOCA and should be pursued.
- This case highlights the importance of CMR in differentiating ischaemic patterns and guiding treatment strategies, significantly improving the patient’s quality of life post-discharge.

## Introduction

Myocardial infarction with non-obstructive coronary arteries (MINOCA) is a diagnostic challenge, affecting up to 10% of patients presenting with acute myocardial infarction.^1^ Despite the absence of significant coronary artery obstruction, these patients face a considerable risk of adverse cardiovascular events.^2^ A stepwise, multimodal diagnostic approach is essential to uncover the underlying pathophysiology and guide appropriate management.^3^

This case report presents a 48-year-old female with MINOCA, highlighting the critical role of advanced imaging modalities, including cardiac magnetic resonance (CMR) and invasive coronary functional testing (CFT). Through a comprehensive evaluation, coronary vasospasm was identified as the culprit mechanism, reinforcing the need for tailored therapeutic strategies. This case underscores the importance of integrating both non-invasive and invasive assessments to achieve an accurate diagnosis, ultimately improving patient outcomes in the complex landscape of MINOCA.

## Summary figure

**Figure:**
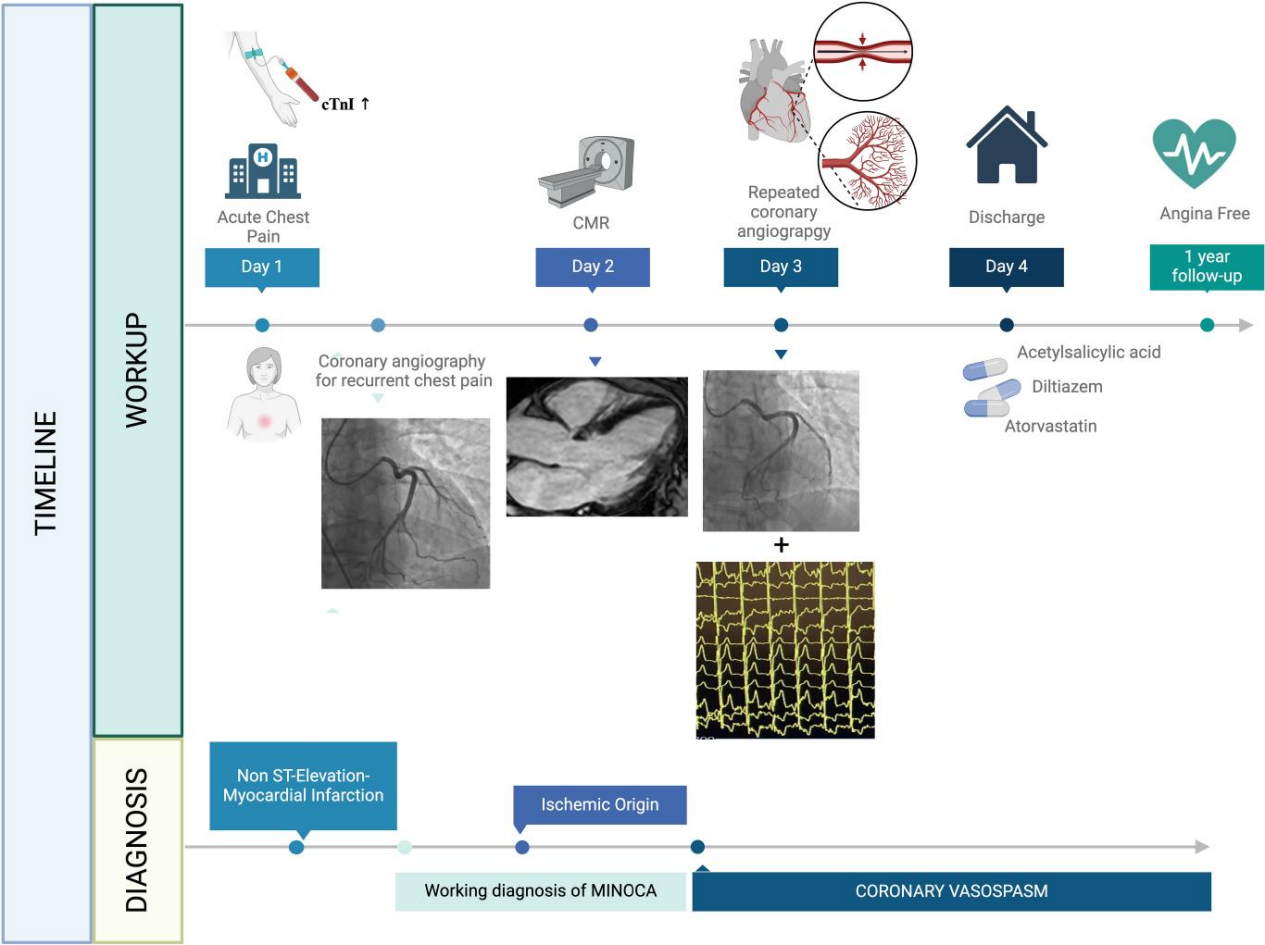


## Case summary

A 48-year-old female patient was admitted to the emergency department with non-ST-elevation myocardial infarction (NSTEMI): (i) clinical presentation with severe chest pain; (ii) normal electrocardiogram (EKG); and (iii) elevation of high-sensitivity troponins at serial determinations (TnI 45 → 1133 pg/mL—upper reference 38 pg/mL). Risk factors included mild dyslipidaemia, glucose intolerance managed with dietary treatment, and active smoking (10 cigarettes/day). The patient had no significant past medical history and was not taking any medication at home. Reported allergies included ceftriaxone and betamethasone.

Upon physical examination, vital signs were within normal range. There were no signs of heart failure, abnormal breath sounds, or abdominal distension. Estimated jugular venous pressure was within range, and no relevant neurological alterations were observed. Blood tests revealed a normal haemogram and creatinine at 0.95 mg/dL (reference range 0.55–1.02 mg/dL). A chest X-ray showed no relevant findings.

Bedside echocardiography revealed mild left ventricular lateral and apical wall hypokinesia with normal global systolic function [left ventricular ejection fraction (LVEF) 55%]. No signs of right ventricular or pressure overload or pericardial effusion were detected.

Given the clinical presentation consistent with NSTEMI, the patient was admitted to the cardiology department following the administration of 250 mg IV acetylsalicylic acid and 80 mg atorvastatin.

**Figure 1:**
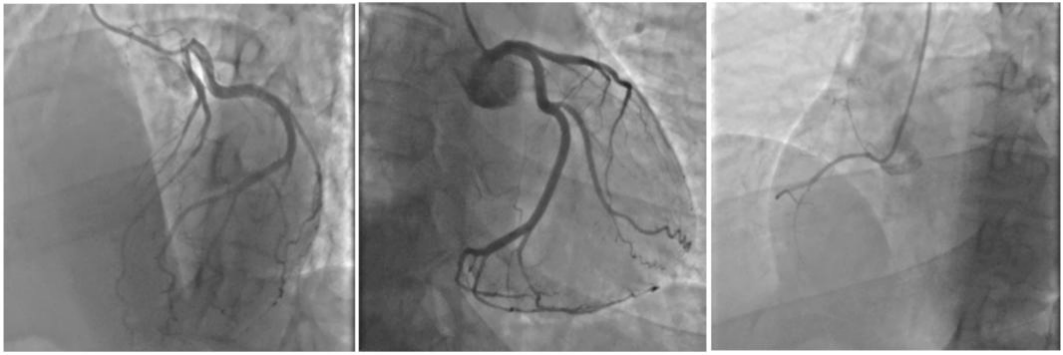
First invasive coronary angiogram. Invasive coronary angiography at admission, showing non-obstructive coronary artery disease. (*Left panel*) Left Anterior Oblique (LAO) cranial view; (*Central Panel*) Right Anterior Oblique (RAO) caudal view; (*Right Panel*) Left Anterior Oblique (LAO) view.

Given the angina recurrence, an urgent invasive coronary angiography (ICA) was performed via the right radial approach on the same day, showing non-obstructive coronary artery disease in the context of left coronary dominance (*Figure 1A–C*). No signs of coronary artery dissection or plaque disruption were present. No peri-procedural complications were observed.

**Figure 2:**
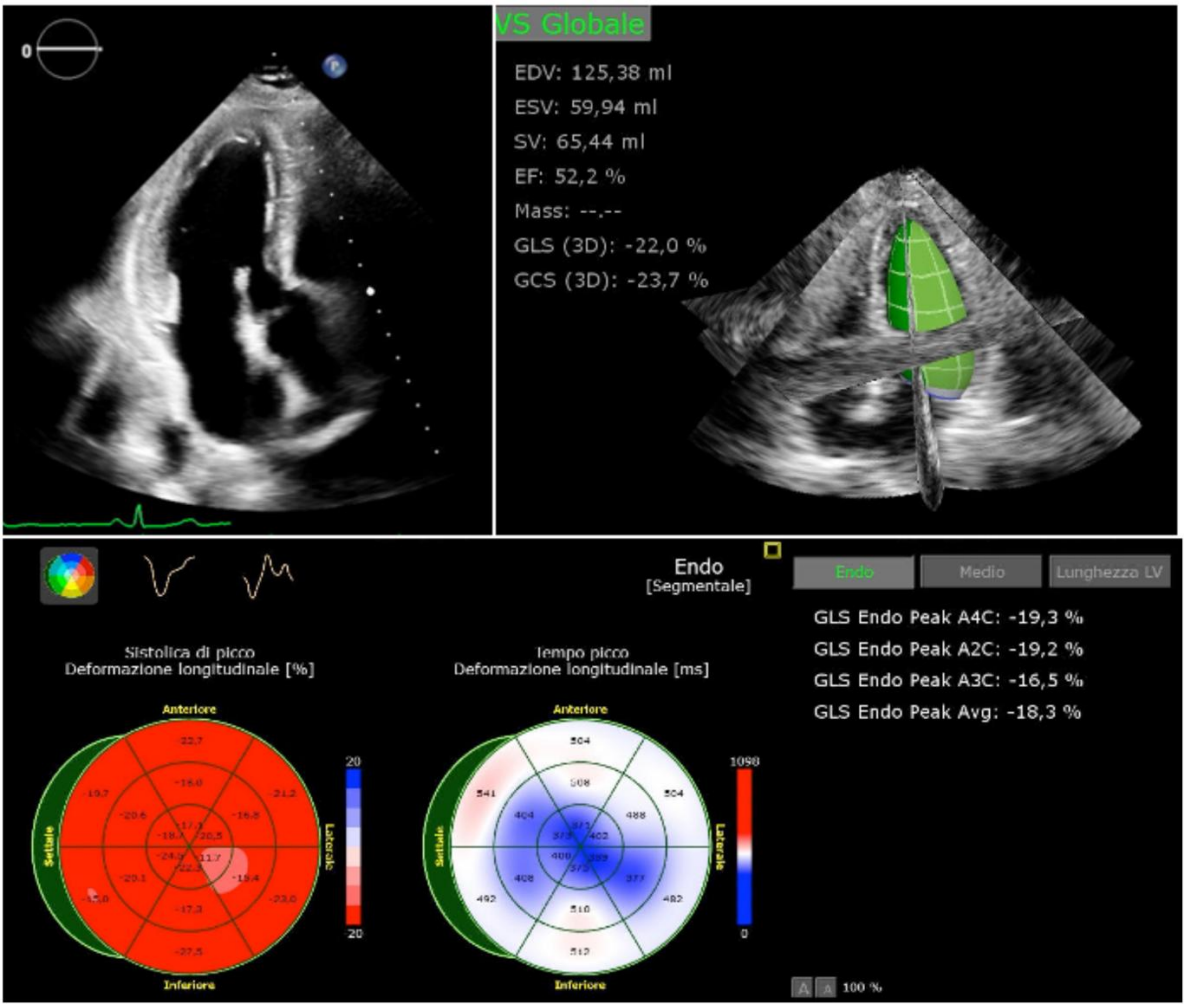
Transthoracic echocardiography. (*Top left panel*) Three-chamber view; (*Top right panel*) 3D echocardiographic ejection fraction and volumes; (*bottom panel*) percentage systolic peak longitudinal deformation index.

**Figure 3:**
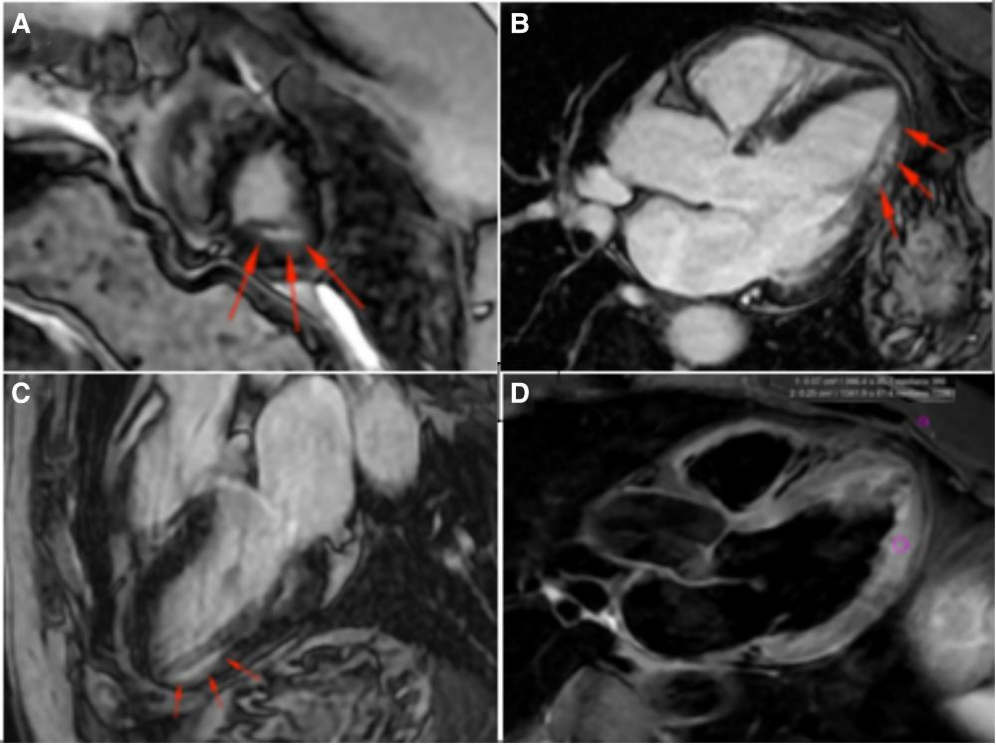
Cardiac magnetic resonance, showing sub-endocardial ischaemic late gadolinium enhancement distribution (red arrows). (*A*) Short-axis view; (*B*) three-chamber axial view; (*C*) three-chamber sagittal view; (*D*) three-chamber time inversion recovery.

During the first day of hospitalization, a progressive rise in myocardial necrosis markers (peak of 3865 pg/mL) was observed. A comprehensive echocardiogram performed on Day 2 revealed normal biventricular dimensions, inferior-lateral mid-apical mild hypokinesia, with normal LVEF (54%) (*Figure 2A*). Myocardial strain analysis appeared to be consistent with regional wall motion abnormalities (*Figure 2C*). No significant valvular heart disease was observed. Due to the working diagnosis of MINOCA, cardiovascular magnetic resonance (CMR) was performed at Day 3, using steady-state free precession, phase contrast, MOLLI, T2 mapping, and late gadolinium enhancement (LGE) sequences. The CMR reported a high signal intensity in the inferior segment on T2-weighted images with sub-endocardial LGE (ischaemic pattern) in the mid-apical posterior-lateral region. These findings excluded myocarditis, cardiomyopathies, and takotsubo syndrome as differential diagnoses (*Figure 3A–D*).

**Figure 4:**
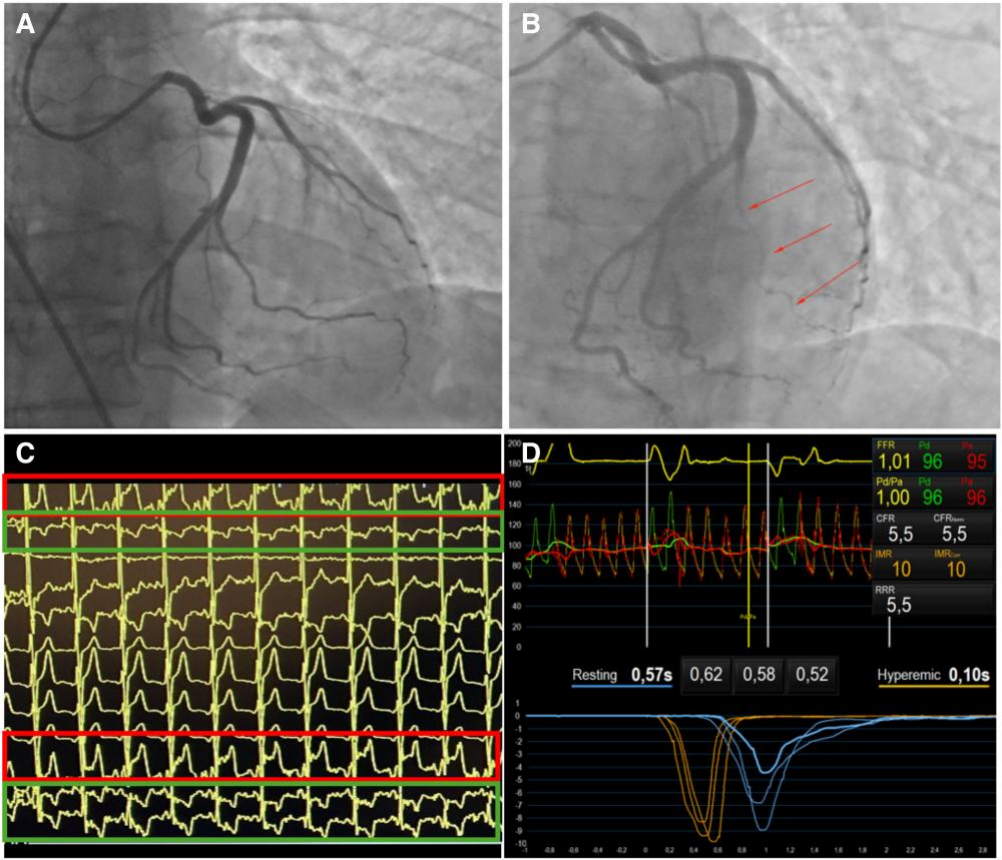
Full-physiology approach during repeat coronary angiography. (*A* and *B*) Invasive coronary angiography showing coronary angiography before and after acetylcholine administration, depicting >90% occlusion of the marginal branch; (*C*) concurrent electrocardiographic findings consistent with high-lateral lead ST-elevation (red boxes) with reciprocal ST-depression changes in inferior leads (green boxes); (*D*) bolus thermodilution showing normal index of microcirculatory resistance (IMR) and coronary microvascular dysfunction.

According to the ischaemic LGE pattern presented at CMR and the recurrence of chest pain with ischaemic EKG changes during hospitalization, a second ICA was performed on Day 4, including CFT. No signs of coronary artery dissection or embolization were confirmed. Bolus thermodilution showed no evidence of coronary microvascular dysfunction (CFR = 5.5 and IMR = 10; *Figure 4A–D*). After adequate time, a step challenge with acetylcholine (Ach) 20 µg→50 µg→100 µg→200 µg protocol was performed. After administration of 50 µg of Ach, severe coronary spasm of a major marginal branch was documented, associated with the occurrence of chest pain and ST-segment elevation in DI and aVL with reciprocal inferior leads changes (*Figure 4B* and *C*). Symptoms rapidly regressed following nitrate administration.

The patient was discharged with diltiazem 60 mg three times daily as the cornerstone vasospasm treatment; statin was added for its known role in improving endothelial function. At 1- and 12-month follow-up visits, the patient showed significant improvement in QoL and angina burden assessed by Seattle Angina Questionnaire (SAQ) 7 questionnaire and EQ5 questionnaire and normal biventricular function at echocardiogram.

## Discussion

Myocardial infarction with non-obstructive coronary arteries affects up to 10% of patients undergoing coronary angiography for acute myocardial infarction.^1,3^ Despite the non-obstructive nature of their coronary arteries, these patients still face a considerable risk of adverse cardiovascular events, with a reported 12-month all-cause mortality rate of 3.4%–4.7%, re-infarction in 2.6%, and up to a quarter experiencing angina within a year, almost mirroring the rates observed in patients with acute myocardial infarction and obstructive coronary artery disease.^3–6^ To ensure an accurate diagnosis of pure ischaemic MINOCA and reveal the pathophysiological mechanism, recent ESC guidelines proposed a practical stepwise approach.^3^ The first step includes a detailed investigation of the patient’s symptoms, medical history, and potential risk factors. When the ICA does not provide a clear cause for MINOCA, additional diagnostic procedures are recommended. These include intravascular ultrasound and optical coherence tomography and CFT.

It is pivotal to understand that the term MINOCA represents a ‘working diagnosis’ including a very heterogeneous population underlying different pathophysiological mechanisms. The ESC guidelines and recent evidence have emphasized the central role of CMR in the diagnostic work-up of MINOCA (Class I level of evidence b recommendation) to differentiate the ‘ischaemic pattern’ from the ‘non-ischaemic’ one to select patients who share the same underlying pathophysiological mechanism. The CMR is effective not only in detecting ischaemic patterns suggesting ‘coronary aetiology’ but also in ruling in/out other alternative diagnoses in about three-quarters of cases.^7^ In this meta-analysis, pure ischaemic MINOCA was confirmed in 1/5th of the patients while reclassification was achieved in 2/3 of the patients.^7^ Crucially, CMR in this context serves not only as an excellent diagnostic tool but also as a predictor of adverse cardiovascular events, mainly affecting patients with an ischaemic pattern.^7,8^

Coronary vasospasm is a frequent cause of MINOCA, with studies indicating that it is responsible in nearly half of the cases when CFT is conducted.^2,9–11^ Importantly, as demonstrated in our clinical cases, side branches may supply large areas of myocardial mass with relevant clinical implications.

The prognosis of MINOCA patients with positive vasomotor tests has been extensively investigated, revealing worse clinical outcomes (in terms of all-cause death, recurrent MI, and need for further ICA) at a median follow-up of 5 ± 3.5 years, compared to those with a negative test.^9^ MINOCA patients with a positive ACh test had a significantly higher incidence of major cardiovascular and cerebrovascular events (MACCE) (22.6% vs. 5.1%, *P* = 0.006), primarily due to an increased rate of hospitalization for unstable angina. These patients also experienced a higher rate of recurrent angina and a lower SAQ summary score at follow-up, indicating a reduced quality of life.^2^ Recent studies have highlighted the complex role of aspirin in MINOCA patients, particularly in cases involving coronary vasospasm.^12^ While aspirin is widely used for its antiplatelet effects, evidence suggests it may exacerbate vasospasm.^13^ A large study has shown that low-dose aspirin might not reduce future cardiovascular events in patients with vasospastic angina.^12^ On the other hand, ACE inhibitors have shown promise in improving endothelial function, which may have beneficial effects in patients with endothelial dysfunction and improving cardiovascular outcomes.^14^ These findings underscore the importance of personalized treatment strategies when managing MINOCA patients with vasospasm in an area still to be unveiled.

Recent studies have highlighted the complex role of aspirin in MINOCA patients, particularly in cases involving coronary vasospasm.^12^ While aspirin is widely used for its antiplatelet effects, evidence suggests it may exacerbate vasospasm.^13^ A large study has shown that low-dose aspirin might not reduce future cardiovascular events in patients with vasospastic angina.^12^ On the other hand, ACE inhibitors have shown promise in improving endothelial function, which may have beneficial effects in patients with endothelial dysfunction and improving cardiovascular outcomes.^14^ These findings underscore the importance of personalized treatment strategies when managing MINOCA patients with vasospasm in an area still to be unveiled.

This case illustrates how combining both non-invasive (CMR) and invasive diagnostic tests (ICA + intravascular imaging and/or CFTs) can result in an accurate diagnosis, revealing the underlying mechanism, which is crucial for determining the most effective treatment strategy.

## Data Availability

The data underlying this article will be shared on reasonable request to the corresponding author.

## Lead author biography

Edoardo Conte is a recognized expert in advanced cardiovascular imaging, with a special focus on the non-invasive study of coronary atherosclerosis, to which he has devoted much of his research. He has also focused on pericardial diseases and sports cardiology. He is a Fellow of the European Association of Cardiovascular Imaging and a member of the Research Committee of the European Association of Preventive Cardiology and the ESC’s Myocardial and Pericardial Diseases Study Group. Main clinical and scientific interests are coronary CT and cardiac MRI, ischaemic heart disease, cardiomyopathies, heart failure, sports cardiology, and pericardial diseases.

## Notes

**Consent:** The authors confirm that a written consent for submission and publication of this case report including images and text was obtained from the patient in line with the COPE guidelines.

**Funding:** None declared.
